# Enhancements and On-Site Experimental Study on Fall Detection Algorithm for Students in Campus Staircase

**DOI:** 10.3390/s25237394

**Published:** 2025-12-04

**Authors:** Ying Lu, Yuze Cui, Liang Yan

**Affiliations:** 1 College of Resources and Environmental Engineering, Wuhan University of Science and Technology, Wuhan 430081, China; luying@wust.edu.cn (Y.L.); 202301704094@wust.edu.cn (Y.C.); 2 Hubei Chemical Safety Association, Wuhan 430060, China

**Keywords:** campus safety, stepping on the stairwell, improved YOLOv7 algorithm, fall detection experiment, stampede prevention

## Abstract

Campus stairwells, characterized by their crowded nature during certain short periods of time, present a high risk for falls that can lead to dangerous stampedes. Accurate fall detection is crucial for preventing such accidents. However, existing research lacks a detection model that balances high precision with lightweight design and lacks on-site experimental validation to assess practical feasibility. This study addresses these gaps by proposing an enhanced fall recognition model based on YOLOv7, validated through on-site experiments. A dataset on campus stairwell falls was established, capturing diverse stairwell personnel behaviors. Four YOLOv7 improvement schemes were proposed, and numerical comparison experiments identified the best-performing model, combining DO-DConv and Slim-Neck modules. This model achieved an average precision (mAP) of 88.1%, 2.41% higher than the traditional YOLOv7, while reducing GFLOPs from 105.2 to 38.2 and cutting training time by 4 h. A field experiment conducted with 22 groups of participants under small-scale populations and varying lighting conditions preliminarily confirmed that the model’s accuracy is within an acceptable range. The experimental results also analyzed the changes in detection confidence across different population sizes and lighting conditions, offering valuable insights for further model improvement and its practical applications.

## 1. Introduction

Stampede accidents are sudden and unpredictable [1], often occurring in crowded settings [2]. On campuses, with their dense student populations and concentrated activities, the risk of stampedes leading to student injuries is heightened [3]. In severe cases, especially in primary and secondary schools, such incidents can result in significant casualties and societal impact. Studies show that out of 40 campus stampedes, 38 occurred in stairwells [4,5], with falls accounting for 27.38% of these accidents, highlighting staircases as high-risk areas and falls as a major contributing factor [6]. Therefore, developing intelligent fall detection systems for campus stairwells is crucial in preventing stampede accidents.

Fall recognition research is divided into wearable device-based and computer vision-based methods [7,8,9]. While wearable devices offer real-time recognition, they are limited by cost, battery life, and missed detections [10,11], making them impractical for large-scale deployment. In contrast, computer vision-based methods, such as skeleton recognition [12,13] and image target detection [14,15], provide higher precision, lower cost, and no reliance on wearable devices, making them the preferred approach. Campus stairwells, with challenges like narrow spaces, debris, low lighting, and high pedestrian flow, complicate fall detection. Skeleton recognition methods, especially those using depth cameras like Kinect, help mitigate lighting issues [16,17,18], but sensor interference can reduce precision. Compared to skeleton recognition, YOLO, known for its speed and precision [19], offers fewer limitations on the number of individuals and is cost-effective, making it ideal for real-time fall detection in high-density environments like campus stairwells.

The compatibility between the dataset and the recognition target directly impacts the performance of object detection algorithms. Current research often uses public datasets for model training. Xu [20] used the Microsoft COCO dataset to train YOLO, analyzing crowd size and speed for stampede monitoring. Ding [21] compared the improved YOLOv4 model with existing models using the Pascal VOC07 + 12 and MS COCO 2017 datasets, finding improved efficiency. Yousef Sanjalawe [22,23] utilized the CUCAFall and DiverseFALL10500 datasets, integrating YOLOv8 with TST and HyperFallNet to enhance fall behavior detection performance. While public datasets offer large sample sizes and rich content, they may not fully address the behavioral diversity and environmental interference in campus stairwell fall detection [24]. Stairwell behavior is diverse, with students engaging in activities such as running or bending, which need to be distinguished from falls. Thus, the dataset must include appropriate negative samples. Additionally, environmental factors like poor lighting and debris must be considered. Therefore, creating a dataset that accounts for both behavioral diversity and environmental conditions in stairwells is essential for effective fall detection using YOLO.

Once an appropriate dataset has been identified, it becomes feasible to establish a deep learning model for detecting falls in campus stairwells. Lin [25] proposed a cloud–fog computing architecture for real-time fall detection, integrating age estimation to improve adaptability and accuracy across age groups in dynamic environments. Despite the significant improvements in detection precision and speed demonstrated by the YOLOv7 algorithm compared to its predecessors at the algorithm level, it employs a large number of standard convolutions, leading to substantial memory consumption. Some studies have recognized this challenge and opted for lightweight models to reduce computational costs and actual running time [26]. However, simple lightweight models inevitably compromise detection precision [27]. Jocher proposed YOLOv5 based on YOLOv4, which is lighter than its predecessor but sacrifices some performance [21]. Since students of different ages may have varying exercise behaviors on campus, Yang [28] used federated learning to enhance CT denoising models, enabling privacy-preserving collaboration and personalization. This method can be applied to the future direction of fall detection in campus stairwells, enabling privacy-preserving distributed training, personalized fall detection, and real-time model updates, thereby enhancing the system’s adaptability and scalability across various campus environments. Overall, there is a need for a lightweight yet high-precision structure to improve and enhance the performance and inference speed of the initial model. Subsequently, numerical experiments can determine the optimal model.

Although numerical experiments offer valuable insights into model performance, they may not fully capture the complexities of real-world conditions, such as low lighting, crowded environments, and dynamic human behavior. A few studies, such as Liao [29], used both numerical and on-site experiments for validation, though their on-site testing involved only one experimenter. Recent work [30] has demonstrated that fall detection accuracy significantly deteriorates under challenging conditions including varying light, crowd density, and occluded body parts. Similarly, David [31] highlights how existing staged datasets often fail to reflect the variability of real-world settings, leading to substantial performance gaps when models are deployed in practical applications. To better assess the model’s real-world performance, an on-site experiment will be conducted to evaluate its practicality, exploring how confidence varies with changes in crowd size and lighting conditions. This will provide valuable insights for improving the fall detection model and adjusting its parameters.

The study improves YOLOv7 by integrating existing modules and adapting it to the complex and dimly lit characteristics of campus stairwells, enabling fast and accurate fall detection in these environments. Additionally, it introduces on-site experiments to examine changes in model confidence under real-world stairwell conditions. The specific work involves establishing a fall dataset tailored to campus staircases, taking into account the behavioral and environmental characteristics of individuals in such settings. Four improved YOLOv7 schemes are proposed, and numerical comparison experiments are conducted to identify the optimal model based on the results. Furthermore, a total of 22 on-site experiments were designed and conducted, including different people groups and luminance groups (422 lux, 14 lux, and 2 lux). These experiments verified the model’s practicality and revealed the confidence variation pattern under different operating conditions, providing a basis for improving the recognition algorithm in the future.

The main contributions of this study are as follows: (1) A campus stairwell fall dataset was established, and through method comparison, the optimal solution for this scenario was identified. By incorporating DO-DConv and Slim-Neck modules, the model reduces memory consumption and enhances processing speed, making it suitable for fall detection. (2) Through comprehensive preliminary verification by field experiments, it was demonstrated that the fall recognition model proposed in this study maintains high precision even in scenarios with small-scale congestion environments and low illumination levels. Furthermore, by analyzing the confidence changes obtained from the on-site experiments, valuable insights are provided for the refinement of further future algorithms and models. This is of great significance for the prevention of falls and stampedes in campus staircases and for improving campus safety.

## 2. Methods

**Figure 1:**
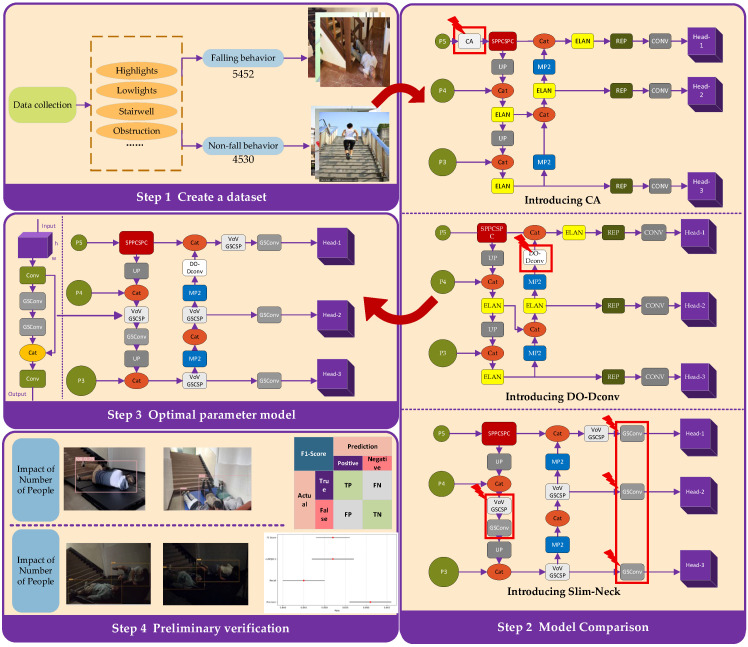
Research overview diagram.

In response to the lack of detection models that balance high accuracy with lightweight design and the absence of on-site empirical validation in existing research, this study first constructs a fall detection dataset specifically for campus stairwells, collecting images that cover various fall postures, lighting conditions, and occlusion scenarios. This approach addresses the poor feature adaptability of general datasets in specific environments. Secondly, four improvements to the YOLOv7 model are proposed, with the goal of optimizing computational efficiency while enhancing detection accuracy, effectively overcoming the original model’s high computational complexity and real-time deployment challenges. Finally, 22 on-site experiments, covering a range of crowd sizes and lighting conditions, were conducted to provide preliminary empirical validation of the optimal model, confirming its reliability in complex real-world environments. The overview is shown in Figure 1.

### 2.1. Establish a Campus Staircase Fall Dataset

Collecting data and images from public channels such as news websites and search engines effectively avoids the problem of visual feature homogenization. The collected data is categorized into unsafe behaviors and unsafe states of matter.

A total of 4436 images were collected, of which 2901 images contained fall behaviors, including three types: forward falls, backward falls, and lateral falls. The criterion for determining a fall behavior is when a person is struck to the ground. A total of 743 images were collected for non-fall behaviors, including running and walking. Since these actions are continuous, and only one frame per action is recorded in the images, the determination of the action is based on its completion. Running is defined as when one leg is raised to half the height of the thigh in an upright position. A total of 792 images were collected for squatting and standing stationary behaviors. Details are shown in Table 1.

When considering environmental lighting, collecting images only in well-lit scenes, such as during the day, may result in poor performance of the trained model. Therefore, images collected should also include scenes with insufficient lighting. A total of 2894 images were collected, including 1034 images of fall behaviors and 1860 images of non-fall behaviors. The presence of obstacles in stairwells, such as debris, can affect detection precision by causing partial occlusion of individuals. As a result, images containing occlusions were also collected, totaling 1877 images. Of these, 742 were fall behaviors, and 1135 were non-fall behaviors.

To align with the study of falls in stairwells, a total of 775 images of individuals falling on stairs were collected during the image gathering process. Details are shown in Table 1.

After establishing the campus staircase fall dataset, it is necessary to annotate the images in the dataset. The annotation software used in this study is Labeling v1.8.6; this software was used to annotate all images, and finally, the campus staircase fall dataset was established.

### 2.2. Comprehensive Improvement Scheme for Campus Staircase Fall Recognition Model

To address the issues of high computational complexity and device requirements in the YOLOv7 algorithm and to further enhance the precision of the model, four improved design schemes for the head network of the initial YOLOv7 model are proposed. Building on previous research, a Coordinating Attention (CA) module is incorporated into the head network to achieve improved detection precision. Introducing DO-DConv instead of standard convolution in the head network can reduce the computational complexity of the algorithm without affecting the extraction strength. Propose a Slim-Neck structural module to reduce model computational complexity and inference time. The head network is further enhanced by combining DO-DConv with Slim-Neck modules. Numerical experiments are conducted to compare the results of these four schemes, ultimately determining the optimized improved model.

#### 2.2.1. Improved YOLOv7 Algorithm Combined with CA

**Figure 2:**
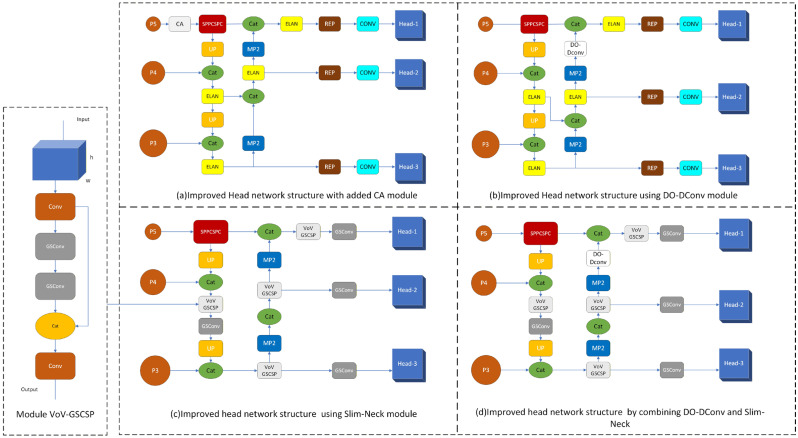
Head network structure diagram of all improvement schemes.

Theoretically, since the location of a fall is only somewhere in the image, it is only necessary to concentrate resources to identify the area in the image so as to improve the precision of fall behavior recognition. Therefore, a coordinate attention mechanism module (CA) that can focus on key objects is introduced [32]. This module embedded the captured location information into the channel attention, which is the encoding process of dividing the channel attention into two one-dimensional features. This enables the long-distance dependencies to be captured in the direction of a single space; accurate location information is also saved in another spatial direction. The resulting feature map is then encoded to form a pair of orientation-perception and location-sensitive feature maps, which can be complementary and applied to the input feature map to strengthen the expression of the attention object [33,34]. The advantage of the CA module is that it makes the model pay more attention to useful channel information. Based on this, this study incorporates CA into the YOLOv7 algorithm to improve recognition precision. When combining CA to improve YOLOv7 algorithm, only the CA module needs to be added to YOLOv7’s head network, and the network structure is shown in Figure 2a.

#### 2.2.2. Improved YOLOv7 Algorithm Combined with DO-DConv

DO-DConv refers to the depth of the hyper parameterized convolutional layers. It is an improvement on the basis of DO-Conv (deep hyperparametric convolutional layer). The principle of DO-Conv is to add an additional deep convolution operation to a common convolution layer, forming an over parameterized convolution layer to replace the CNN ordinary convolution layer, thereby greatly improving the performance of CNN convolution layers [35]. When reasoning, DO-Conv can be converted to traditional convolution operations, so using DO-Conv instead of traditional convolution in a network does not increase computational requirements [21]. Similarly, DO-DConv is obtained by adding an additional depth convolution operation to the depth convolution [27].

The calculation process of DO-Conv can be divided into two categories: Feature Composition and Kernel Composition. This improvement adopts Kernel Composition.

The calculation process of Kernel Composition can be expressed as

(1) $$\mathrm{Kernel}\;\mathrm{Composition}\left\{\begin{array}{l} W^{\prime }=D^{T}\circ W:D_{mul}\times \left(M\times N\right)\times C_{in}\times C_{out}\\ O=W^{\prime }*P>:C_{out}\times C_{in}\times H\times W\times \left(M\times N\right) \end{array}\right.$$

The first step is to use a trainable kernel ***D^T^*** to transform ***W***, calculate the product of two weights, and generate a new weight $W^{\prime }$:

(2) $$W^{\prime }=D^{T}\circ W$$

In the formula, $D^{T}\in R^{D_{mul}\left(M\times N\right)\times C_{in}}$ is the transpose of $D\in R^{\left(M\times N\right)\times D_{mul}\times C_{in}}$.

***H***—The height of the feature map;

***W***—The width of the feature map.

Step 2 is to use the new weight $W^{\prime }\in R^{D_{mul}\times \left(M\times N\right)\times D_{in}\times C_{out}}$ to perform traditional convolution on the input feature P, and output the final result **O**:

(3) $$O=W^{\prime }*P$$

Therefore, the calculation process of deep hyperparametric convolution can be expressed as

(4) $$O=\left(D,W\right)⊙P=W*\left(D\circ P\right)=\left(D^{T}\circ W\right)*P$$

In this formula, “☉”represents the DO-Conv operation.

Following the same principle, DO-DConv can also be calculated using two mathematically equivalent methods:

(5) $$O=\left(D,W\right)•P=W*\left(D\circ P^{\prime }\right)=\left(D^{T}\circ W^{T}\right)\circ P$$

In this formula, “•” represents the DO-DConv operation.

In the improved YOLOv7 algorithm combined with DO-DConv, depthwise-separable convolutions (DO-DConv) replace the standard convolutional layers in the YOLOv7 head to enhance computational efficiency while preserving performance. Specifically, the 3 × 3 convolutional layers in the head are substituted with depthwise-separable convolutions, where the depthwise convolution uses a 3 × 3 kernel for feature extraction, followed by a 1 × 1 pointwise convolution to combine the extracted features. This modification reduces the number of parameters by utilizing a more efficient convolution technique, while still enabling the model to learn rich feature representations. The final output layer also employs a 1 × 1 depthwise-separable convolution, ensuring to accelerate both training and inference while maintaining or improving detection precision. The network structure of this improved algorithm is shown in Figure 2b.

#### 2.2.3. Improved YOLOv7 Algorithm Combined with Slim-Neck

The lightweighting of models is of great significance for their operational efficiency and wide application. One of the current methods for reducing computational costs is through depthwise-separable convolution (DSC). The DSC method can effectively reduce model parameters and the number of FLOPs. However, when using the DSC method for computation, the channel information of the input image is separated, which can result in the model’s feature extraction and fusion capabilities being significantly inferior to standard convolution (SC). In order to make the output results of DSC as close as possible to SC, Li et al. [36] proposed a new method called GSConv, which completely mixes the information generated by SC (channel dense convolution) into the output of DSC (channel sparse convolution), reducing model complexity while maintaining accuracy. And a new design paradigm, Slim-Neck, is proposed, which is a lightweight Neck layer that reduces model complexity while maintaining model accuracy [35].

Based on the characteristics of GSConv [36], the head network can be improved using the following two approaches:

(1) Replace SC with the lightweight convolution method GSConv. The calculation cost of GSConv is only 60% to 70% of that of SC.
(2) The cross-stage partial network (GSCSP) module VoV-GSCSP is designed using a one-time aggregation method, which reduces the complexity of computation and network structure while maintaining sufficient precision, as shown in Figure 2. Usually, to effectively reduce FLOPs, VoV-GSCSP modules can be used to replace the ELAN and REP modules in the Neck layer [37].

In the improved YOLOv7 algorithm, GSConv is applied to replace the standard 3 × 3 convolutional layers in the Neck. Additionally, VoV-GSCSP is utilized in the Neck and Transition layers, optimizing cross-stage feature aggregation and reducing computational complexity. These modifications enhance the efficiency of feature processing while maintaining or improving the overall performance of the model by reducing FLOPs and the number of parameters, as shown in Figure 2c.

#### 2.2.4. Improved YOLOv7 Algorithm Combining DO-DConv and Slim-Neck

This study attempts to combine the advantages of high precision of DO-DConv and lightweight Slim-Neck, aiming to achieve model lightweight while improving detection precision. The network model is shown in Figure 2d.

### 2.3. Numerical Experiments

#### 2.3.1. Numerical Experiment Purpose and Environment

In order to explore whether the improved model combines the expected lightweight and higher detection precision, comparative experiments were conducted on the four improved model schemes mentioned above, and the optimal yolov7 improved model was obtained. We used precision, recall rate, mAP@0.5, mAP@0.5:0.95, and F1 score to comprehensively evaluate the performance of the model, and GPU consumption, Parameter quantity, GFLOPs, and training duration to evaluate the model complexity. The numerical comparison experimental scheme is shown in Table 2.

To comprehensively evaluate the model’s performance, the study uses the following classification and detection metrics [22].

***Precision (P):***

(6) $$Precision=\frac{TP}{TP+FP}$$

***TP*** is the number of true positives and ***FP*** is the number of false positives.

***Recall (R):***

(7) $$Recall=\frac{TP}{TP+FN}$$

***FN*** denotes false negatives.

***F1-Score***:

(8) $$F1\text{-}\mathrm{Score}=\frac{2•Precision•Recall}{Precision+Recall}$$

Mean Average Precision at IoU 0.5 (***mAP@0.5***):

(9) $$\mathrm{mAP}@0.5=\frac{1}{N}\sum_{i=1}^{N}AP_{i}(\mathrm{at}\;\mathrm{IoU}\geq 0.5)$$

***AP_i_*** is the average precision for class ***I*** and ***N*** is the number of classes.

Mean Average Precision at IoU 0.5:0.95 (***mAP@0.5:0.95***):

(10) $$\mathrm{mAP}@\left[0.5:0.95\right]=\frac{1}{10}\sum_{j=0}^{9}mAP@IoU_{0.5+0.05j}$$

Frames Per Second (***FPS***):

(11) $$\mathrm{FPS}=\frac{Total\;number\;of\;processed\;frames}{Total\;inference\;time\left(seconds\right)}$$

Hardware equipment for the experiment: one host computer with an 11th Gen Intel (R) Core (TM) i7-11800H @ 2.30 GHz CPU; the GPU is NVIDIA GeForce RTX 3060 (Laptop); the GPU has a graphics memory capacity of 6 GB; the memory consists of two Kingston DDR4 3200 MHz 8 GB; the hard drive is Crucial MX500 1 TB.

Experimental software environment: Windows 10 operating system; Visual Studio 2022; Python 3.8; Pytorch 1.8.0; Cudatoolkit 11.3; CuDNN v8.5.0.

#### 2.3.2. Model Training

The algorithm in this article is implemented in the Pycharm integrated software, using Python 3.8 as the programming language and Python 1.8.0 as the framework for deep learning. The image input resolution was fixed at 640 × 640 pixels. Using the YOLOv7.pt pre-training model for training, the training set, test set, and validation set were divided into 7:2:1 with preserved class distribution. Seventy percent of the dataset is allocated for training, allowing the model to learn patterns and features from diverse scenarios. Subsequently, twenty percent of the data is used for validation, facilitating model fine-tuning and hyperparameter adjustment to mitigate overfitting. The remaining ten percent is reserved for testing, ensuring that the model’s performance is evaluated on unseen data, thereby providing a reliable assessment of its generalization capability. The dataset does not include on-site experiment images. On-site experiment personnel only validated the method. Due to hardware limitations, the default value in batch size should be set to 2, and the original and improved YOLOv7 models were trained separately. In this study, a learning rate of 0.005 is used to mitigate the impact of a batch size of 2, which could otherwise reduce the model’s convergence and the stability of the learning process. The K-means clustering algorithm is additionally employed to predefine anchor frames during model training. These strategies facilitate more effective model training and parameter optimization, thereby enhancing the model’s detection precision and robustness.

The loss trends of each model and the total loss trend of the training set obtained after training are given in Section 3.1. The loss function is given in Equations (12) and (13), where ***C*** denotes the minimum convex enclosing box of the predicted bounding box (***A***) and the ground-truth bounding box (***B***). The NMS threshold is set to 0.5.

(12) $$GIoU=IoU-\frac{\left|C/(AUB)\right|}{\left|C\right|}$$

(13) GIoUloss=1−GIoU

### 2.4. Preliminary Validation

#### 2.4.1. Preliminary Validation Purpose and Scheme

To recreate a realistic scenario, the experiment was conducted in the stairwell of a school building, and the similarities to the dataset were minimized. Before the experiment begins, place two protective pads at the staircase for the experimenter to simulate falling protection. Place a camera at a distance of 1 m from the side of the protective pad in a 45° direction and use a tripod to fix the camera at a height of about 1 m from the ground. After the experiment began, the experimenters performed three actions: forward fall, backward fall, and lateral fall according to different group numbers, and each action was completed ten times.

(1) Experimental groups with different numbers of people.

Under sufficient lighting conditions (illumination of 422 lux), considering the influence of the number of participants on the recognition results, a total of three participants participated in the experiment. The three participants had heights of 172 cm, 161 cm, and 155 cm, respectively. The specific implementation scheme is shown in Table 3. Considering the limited space in the stairwell and the safety of the experimenters, the three-person experiment was conducted under the scenario of simultaneous forward falls only.

(2) Different experimental groups under different luminance conditions.

To restore the campus staircase scene and further verify the effectiveness of the model under different environmental brightness, experiments were designed to be conducted under three environmental brightness conditions: sufficient lighting, weak lighting, and extreme weak lighting. The corresponding lighting levels were 422 lux, 14 lux, and 2 lux, respectively. Only a two-person experiment was conducted. The specific implementation scheme is shown in Table 4.

Finally, on-site experiments for non-fall behaviors are conducted according to the experimental groups mentioned above, providing an indirect validation of the reliability of fall behavior detection results.

#### 2.4.2. Experimental Software

**Figure 3:**
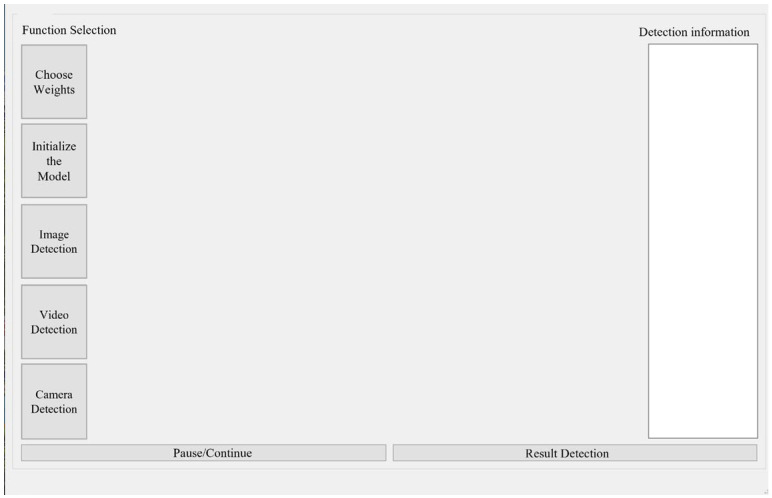
Object detection tool interface.

Based on the optimal model parameters obtained from numerical experiments, the existing software was optimized. The software completed the overall system design through an interface development program in Python 3.8, as shown in Figure 3. The interface can use three methods: image, video, and camera detection. To ensure the real-time recognition of objects, this study chose to use camera detection to identify falls in stairwells.

#### 2.4.3. Experimental Process

(1) Open the object detection tool, click ‘Select Weights’, and select the best trained model, which is best. pt.
(2) Click on ‘Initialize Model’. When ‘Model loading completed’ appears, proceed to the next step.
(3) Click on ‘Camera Detection’.
(4) Participants start the experiment, and each experimental scheme is conducted 10 times.
(5) After completing the action, click ‘End Detection’. Repeat steps 3 and 4 until all participants have completed the corresponding number of groups in the experiment.

## 3. Results

### 3.1. Numerical Experimental Results

**Figure 4:**
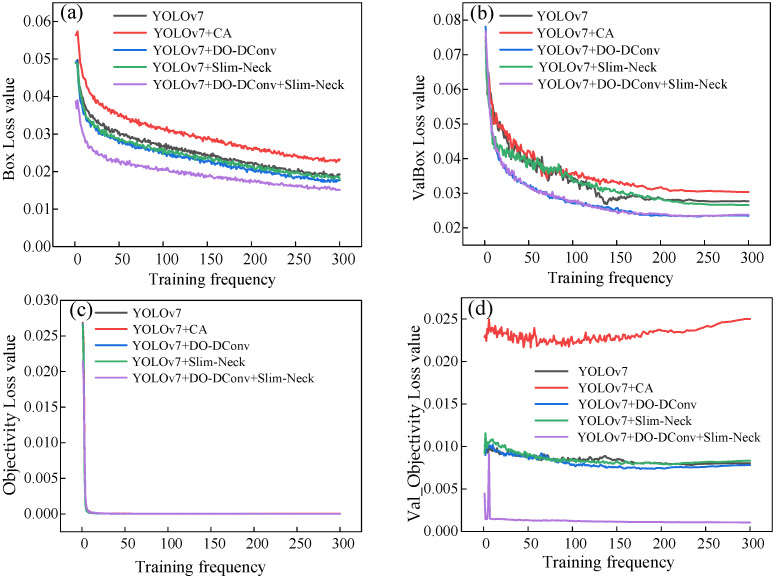

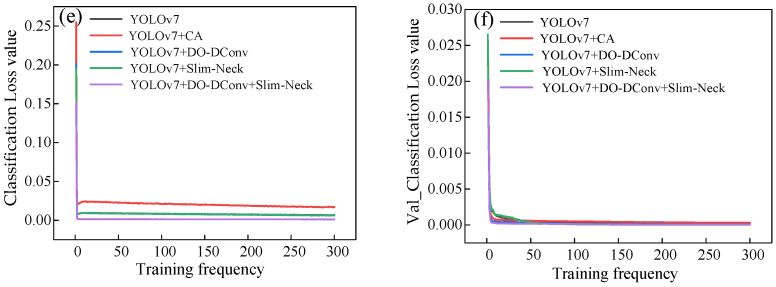
Trend graphs for each loss component: (**a**) training set bounding box loss (Box); (**b**) validation set bounding box loss (Val_Box); (**c**) training set objectness loss (Objectness); (**d**) validation set objectness loss (Val_Objectness); (**e**) training set classification loss (Classification); (**f**) validation set classification loss (Val_Classification).

**Figure 5:**
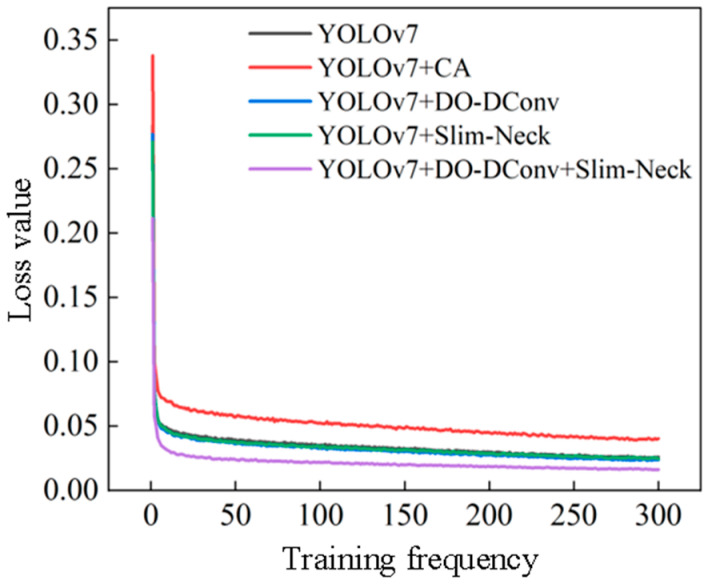
Overall loss trend of the training set.

Figure 4 depicts the loss trends for the YOLOv7 model and each improved model during training, along with the overall loss trend for the training set. The overall loss trend of the training set is shown in Figure 5, illustrating the change in the total loss throughout the training process. Specifically, Figure 4a–f represent the loss trends for the training set bounding box loss (Box), validation set bounding box loss (ValBox), training set objectness loss (Objectivity), validation set objectness loss (Val_Objectivity), training set classification loss (Classification), and validation set classification loss (Val_Classification). In general, all the loss curves show a downward trend, eventually stabilizing.

**Figure 6:**
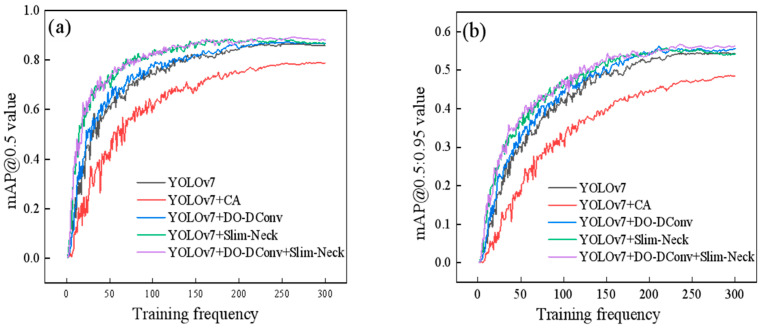
Comparison of the training process variation curve of average detection precision (mAP): (**a**) mAP@0.5; (**b**) mAP@0.5:0.95.

Figure 6 shows a comparison of the variation curves of the mAP @0.5 and mAP @0.5:0.95 during training. From the graph, it can be intuitively seen that the mAP value of the YOLOv7 + CA model has always been the lowest in 300 rounds of training, and the detection precision is lower than the initial YOLOv7 model. This improvement scheme is not ideal. Before about 200 rounds of training, the mAP values of the YOLOv7 + Slim-Neck model and the YOLOv7 + DO-Dconv + Slim-Neck model were the highest and relatively close but were later lower than the mAP values of the YOLOv7 + DO-Dconv + Slim-Neck model. Overall, the YOLOv7 + DO-Dconv + Slim-Neck model has higher detection precision.

Table 5 presents indicators that reflect the size and complexity of the model. The smaller the parameters of these indicators, the lighter the model becomes. From the table, it can be intuitively seen that the YOLOv7 + DO-Dconv + Slim-Neck model has the lowest parameter of each indicator, indicating that the improved model has the best lightweight effect.

### 3.2. Preliminary Validation Results

(1) Different groups with different numbers of people.

**Figure 7:**
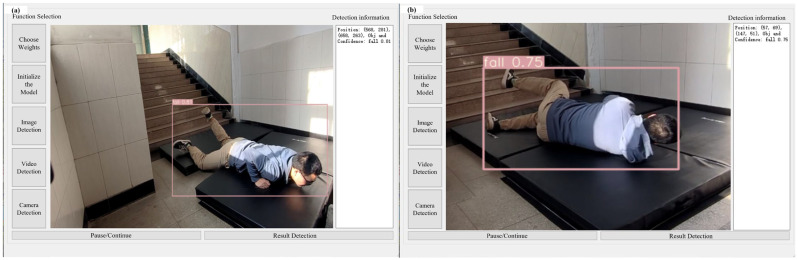

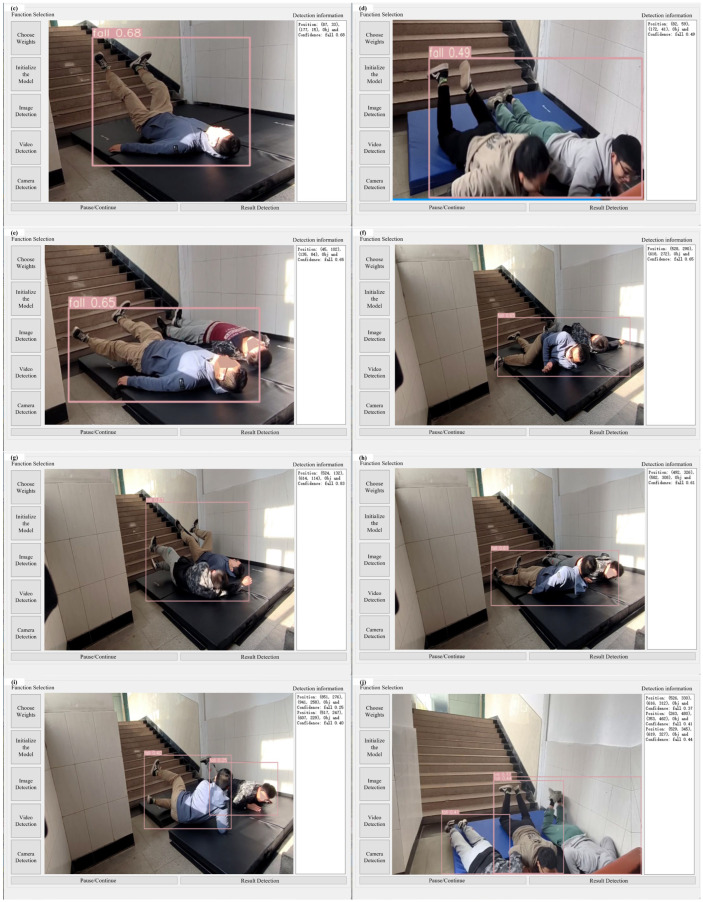
Example of fall detection results for varying numbers of people under sufficient lighting (422 lux): (**a**) experimental condition 1; (**b**) experimental condition 2; (**c**) experimental condition 3; (**d**) experimental condition 4; (**e**) experimental condition 5; (**f**) experimental condition 6; (**g**) experimental condition 7; (**h**) experimental condition 8; (**i**) experimental condition 9; (**j**) experimental condition 10.

According to the scheme in Table 3, experiments were conducted on three groups of falls, and the results are shown in Table 6. Figure 7 shows an example of the experimental interface for a certain frame in groups with different numbers of people.

(2) Different luminance groups.

**Figure 8:**
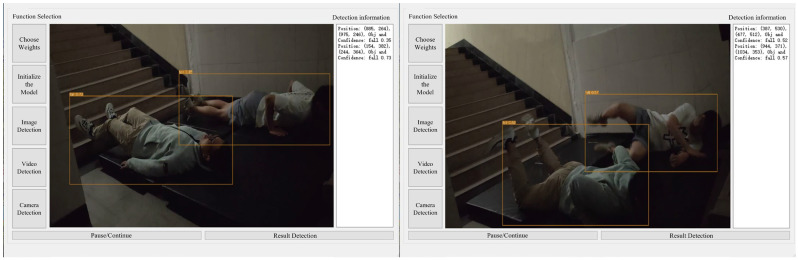
Target detection and identification results of low-light group (14 lux).

**Figure 9:**
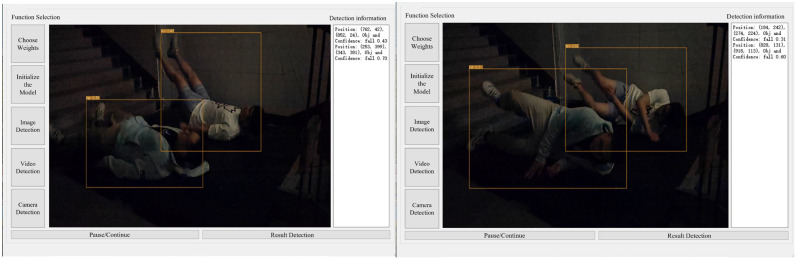
Target detection and identification results of the 2 lux group.

Experiments were conducted according to the protocol outlined in Table 4, with the results presented in Table 7. Figure 8 and Figure 9 provide examples of the experimental interface under ambient illumination of 14 lux and 2 lux, respectively, at a specific frame.

The results of the non-fall detection experiment are shown in Table 8. Overall, the model performs well for stationary actions such as standing and squatting, exhibiting low false alarm rates and high detection precision. However, its performance is slightly less effective for dynamic actions like walking and running, which are associated with higher false alarm rates and a slight reduction in precision. The results from the non-fall sequence testing confirm that the model is not only effective in detecting falls but also highly accurate in minimizing false alarms for various normal activities. The False Alarm Rate (***FPR***) is given by the formula in Equation (14).

(14) $$\mathrm{FPR}=\frac{FP}{FP+TN}$$

## 4. Discussion

### 4.1. Performance Comparison of the Different Models

To validate the superior performance of the improved YOLOv7 model, the study compared its performance metrics with those of YOLOv4-tiny, YOLOv5, YOLOv7, YOLOv8n, and the proposed YOLOv7 improvements. The results are presented in Table 9. YOLOv7 + DO-DConv + Slim-Neck outperforms YOLOv4-tiny, YOLOv5, and YOLOv8n in terms of detection precision, speed, and efficiency. It achieves the highest precision (88.03%), mAP@0.5 (88.10%), and F1 score (0.86), demonstrating superior performance in both object detection precision and model consistency. Although its recall rate (82.32%) is slightly lower than that of YOLOv4-tiny and YOLOv7, it maintains a competitive FPS (114 FPS), similar to YOLOv8n (113 FPS). This combination of high precision and computational efficiency positions YOLOv7 + DO-DConv + Slim-Neck as the most balanced and effective model for practical deployment among the compared algorithms.

From the data in the table, results can be concluded that YOLOv7 + DO-DConv + Slim-Neck performed the best overall, improved the improvement precision by 6.44% compared to YOLOv7, reduced the recall rate by 1.49%, and the average detection precision increased by 2.41% and 1.93%, respectively, while the F1 score increased by 0.03. The overall performance of YOLOv7 + DO-DConv and YOLOv7 + Slim-Neck is relatively similar. The overall performance of YOLOv7 + CA was the worst, with each indicator being significantly backward from YOLOv7, and the comprehensive performance was not as good as the original YOLOv7 model. In addition, from Figure 4 and Figure 5, although YOLOv7 + DO-DConv + Slim-Neck is close to the curve of the model than the other four in some figures, it is significantly lower than the other models in Figure 4a (loss of bounding box in the training set) and Figure 4d (target loss map in the validation set), and overall superior to the other models.

**Figure 10:**
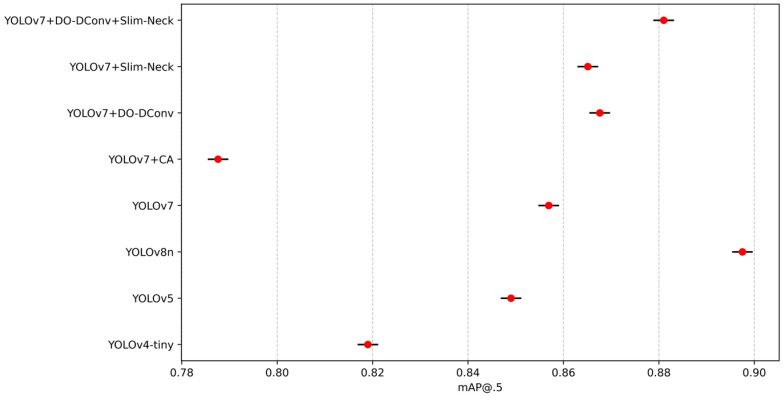
The 95% confidence intervals for mAP@0.5.

**Figure 11:**
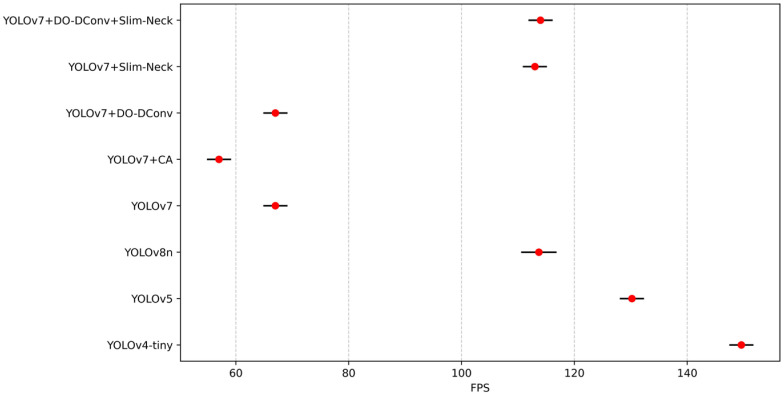
The 95% confidence intervals for FPS.

**Figure 12:**
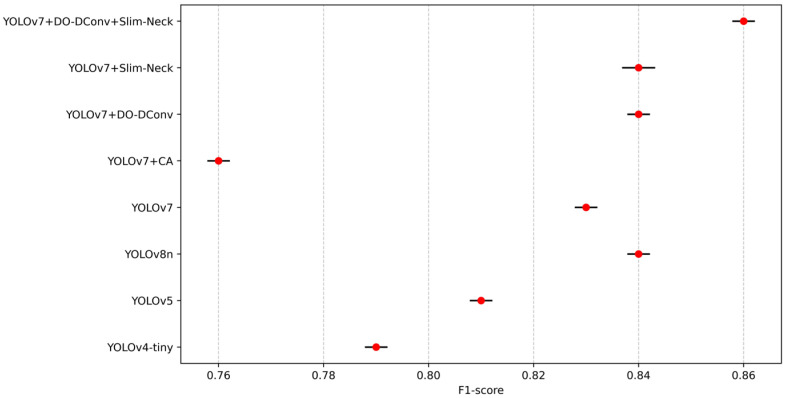
The 95% confidence intervals for F1-score.

**Figure 13:**
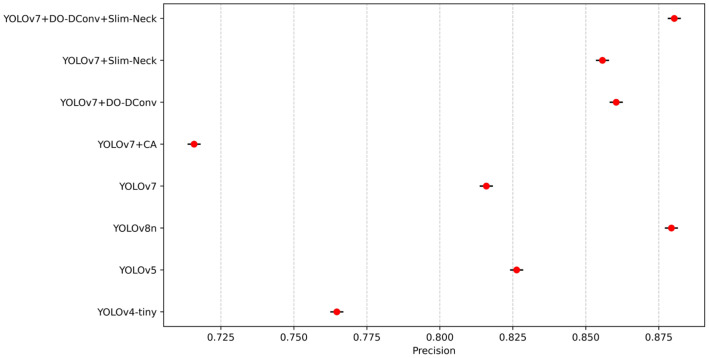
The 95% confidence intervals for precision.

**Figure 14:**
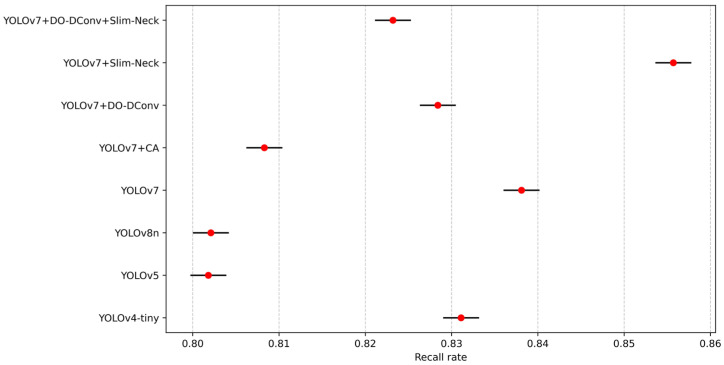
The 95% confidence intervals for recall rate.

To validate the robustness of the performance differences, statistical tests were conducted to determine whether the improvements in mAP@0.5, F1 score, and FPS are statistically significant and consistent across different runs. Each model was evaluated through ten independent runs with random seeds [2010, 2011, 2021, 2033, 2039, 2047, 2057, 2068, 2074, 2088] under the same training settings with an epoch of 300 using SGD with momentum set to 0.937 and weight decay of 0.0005. To enhance generalization and prevent overfitting, the dataset was augmented with random rotations (±10°), horizontal flipping, and scaling variations to simulate real-world perspective changes. Random masking was applied to small regions to train the model to handle occluded individuals. Figure 10, Figure 11, Figure 12, Figure 13 and Figure 14 present the 95% confidence intervals for all models across the three core metrics. The variability observed across all metrics is minimal, with standard deviation values not exceeding 0.5%.

From the parameters of the size and complexity of the reaction model in Table 9, the YOLOv7 + DO-DConv model combined with DO-DD-DConv module reduced the number of parameters and the number of floating point operations per billion (GFLOPs) compared with YOLOv7, but the decrease was slightly lower, by 545,792 and 0.5, respectively, while the GPU consumption increased by 0.01 G, and the training time did not change significantly. After combining the Slim-Neck module, all indicators of the YOLOv7 + Slim-Neck model were greatly reduced, with a GPU consumption and parameter number of 2.48 G and 34,246,516, respectively. The training duration and GFLOPs were 14 h and 38.5, respectively. After simultaneous improvement with DO-DConv and Slim-Neck, the training speed of the YOLOv7 + DO-DConv + Slim-Neck model was further improved, optimal the five sets of models, with GPU consumption, number of parameters, GFLOPs, and training duration of 2.46 G, 33,113,460, 38.2, and 13 h, respectively. The YOLOv7 algorithm combined with CA significantly increased the YOLOv7 algorithm in GPU consumption, parameter number, GFLOPs and training time, with 2.54 G, 37,969,872, 119.7, and 21 h, respectively, which showed the least ideal performance in these five types of models.

In conclusion, the YOLOv7 + CA model performs the most unsatisfactory. The analysis is that the attention mechanism focuses too much on a certain part of the picture, making it unable to accurately locate or mislocate the identified target, while wasting more time. The detection precision of the YOLOv7 + DO-DConv model is significantly improved compared with the YOLOv7 model. Although the training speed of the model is improved after using the depth overparametrization of the depth convolution to a certain extent, the improvement effect is not obvious. The YOLOv7 + Slim-Neck model greatly improves the training speed, but the detection performance improves less. However, the YOLOv7 + DO-DConv + Slim-Neck model proposed based on the advantages of the two has improved the detection performance and training speed and has the advantages of high precision and lightweight.

### 4.2. Analysis of the Impact of Population Changes

**Figure 15:**
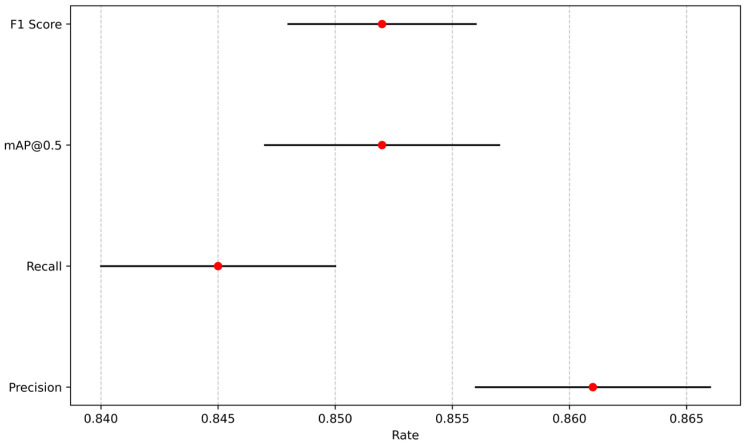
The 95% confidence intervals for impact of different numbers of people.

To better illustrate the impact of the number of people on recognition performance, recognition metrics were collected for each experimental group at 1, 2, 3, and 4 s after the participants hit the ground. Precision, recall, mAP@0.5, and the standard deviation of F1 score were all less than 0.5%. The 95% confidence intervals shown in Figure 15 indicate that the model is stable and reliable, with minimal variations between runs. In the single-person experiment, the confidence score continuously increased throughout the fall, indicating high accuracy in single-person recognition. Table 10 shows the data results of a certain experiment. In the multi-person scenario, for example, in the case of two people falling backward, the confidence scores in experiments 4, 5, 6, 8, 9, and 10 showed a decrease at the 2nd and 3rd seconds, before recovering to higher levels after the 4th second. Analysis reveals that the relative positions of Experimenters A, B, and C with respect to the camera vary, and thus Experimenter A may block the view of Experimenters B and C during the fall, causing interference in the model’s recognition of B and C. This issue can be addressed by adjusting the camera position. Additionally, a low camera frame rate can result in motion blur during the fall, affecting recognition accuracy. This can be optimized by using a higher-performance camera. Although the model exhibited temporarily lower confidence scores in the multi-person fall scenario, the recognition accuracy remained within an acceptable range, indicating that the model is capable of recognizing multiple people falling simultaneously.

### 4.3. Analysis of the Impact of Changes in Light Intensity

**Figure 16:**
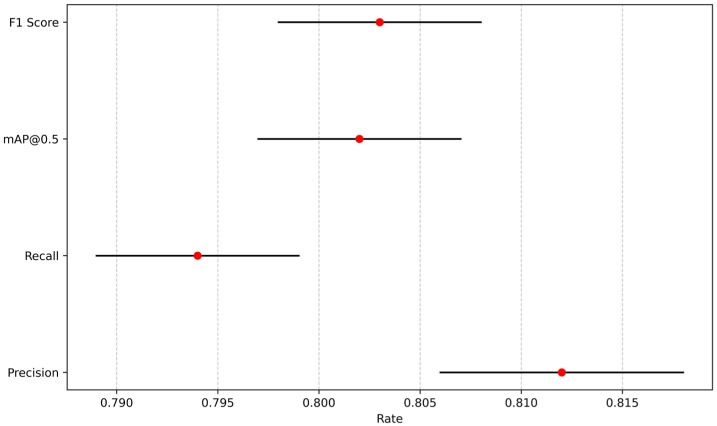
The 95% confidence intervals for impact of 14 lux.

**Figure 17:**
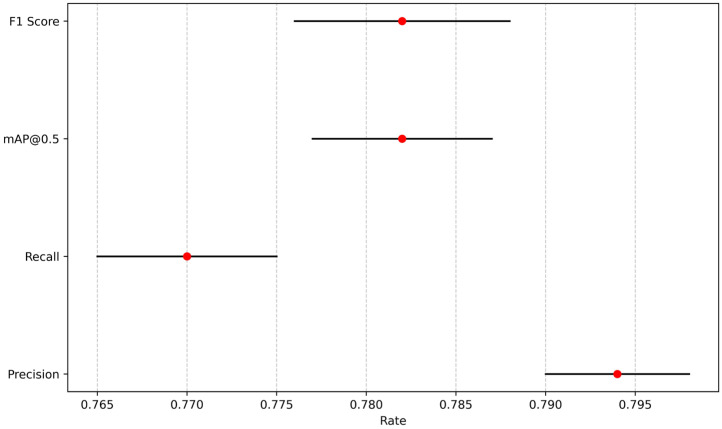
The 95% confidence intervals for impact of 2 lux.

Precision, recall, mAP@0.5, and the standard deviation of F1 score were all less than 0.5%. The 95% confidence intervals shown in Figure 16 and Figure 17 indicate that the model is stable and reliable, with minimal variations between runs. The model does not overfit or underfit any specific part of the data, demonstrating good generalization ability. In Table 11, the confidence of the low-light group (14 lux, 2 lux) was somewhat comparable to the full-light group (422 lux), but the decrease was lower. Although there are very few frames with a confidence level of 0, overall indicator performance is at an acceptable level. This indicates that the model can meet the recognition requirements for stairwells under different luminance conditions.

In conclusion, the proposed fall recognition model based on improved YOLOv7 can not only balance the detection precision and lightweight but also maintain high detection precision under different luminance conditions and multiple people.

## 5. Conclusions

This paper presents an enhanced model for fall detection in campus staircases based on YOLOv7, validated through on-site experiments, which also reveal key patterns in the model’s confidence levels under varying conditions. Initially, a dataset tailored for campus stairwell fall cases was established. Four improvement schemes were proposed and numerical comparison experiments showed that the combination of DO-DConv and Slim-Neck yielded the optimal model, balancing high detection precision and lightweight design. A total of 22 on-site experiments were conducted, with preliminary validation showing that all metrics remained within an acceptable range under various conditions. Confidence variation analysis revealed that temporary decreases in confidence, including brief drops to zero, reflect the model’s momentary failure under extreme challenge conditions, such as severe occlusion and motion blur. Since a fall is a continuous process, these fluctuations do not hinder the model’s ability to accurately detect fall events. Furthermore, the observed patterns of confidence variation provide valuable insights for improving future models and enhancing practical applications. Overall, the proposed model is capable of accurately detecting falls in low-light and small-scale congestion environments, offering significant value in preventing stampede accidents in campus staircases.

## 6. Limitations and Future Works

This study demonstrates effective fall detection in typical teenage student scenarios but has limitations when applied to young children. Future research will enhance the model’s robustness by incorporating more diverse datasets and adjusting detection thresholds to ensure reliable fall detection among people of all ages and ability levels on campus.

Although the preliminary validation shows the scheme’s practicality, some cases still result in a confidence value of zero, affecting detection performance. To address this, a buffer window will be introduced in the alarm triggering mechanism. If confidence drops at a critical moment, the system will trigger the alarm after confirming the event’s continuity, providing a time window for fall detection.

While the current performance is acceptable, this study provides only preliminary validation based on small-scale pedestrian traffic and does not account for other factors such as camera angles, heights, distances, student attire, backpack types, pedestrian flow, and staircase usage during peak hours. Future work will update the dataset to incorporate these variables and conduct further validation experiments to thoroughly evaluate the model’s performance.

## Figures and Tables

**Table 1:** Example of action and environment classification for campus staircase fall dataset.

Act	Examples of Partial Dataset Images				
Falling	Falling forward	Falling backward		Falling sideways	
	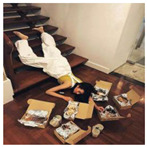	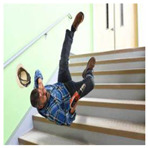		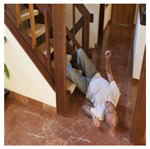	
Non falling	Running	Walking	Standing		Squatting
	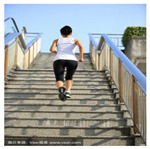	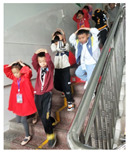	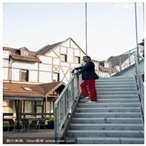		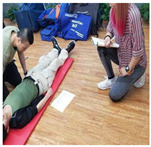
Obstruction	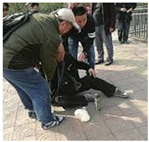	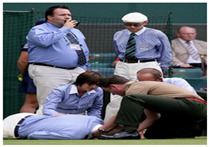		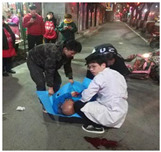	
Actions in the Dark	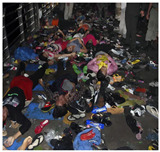	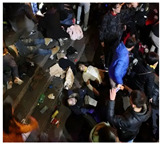		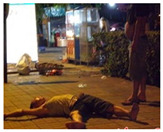	
Falling down the stairs	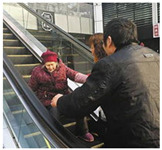	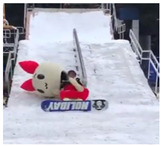		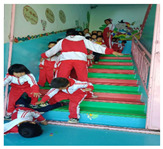	

**Table 2:** Numerical comparison experimental scheme.

Serial Number	Improvement Scheme	Dataset	Performance Index
1	YOLOv7 + CA	Self-Constructed Dataset	Precision, recall rate, mAP@0.5, mAP@0.5:0.95, F_1_ score Model complexity: GPU consumption, Parameter quantity, GFLOPs, training duration
2	YOLOv7 + DO-DConv		
3	YOLOv7 + Slim-Neck		
4	YOLOv7 + DO-DConv + Slim-Neck		

**Table 3:** Experimental scheme for different populations under adequate light (422 lux).

Illuminance	Experimental Group Number	Experimental Group Number	Forward Fall	Backward Fall	Sideways Fall	The Number of Experiments
422 lux	A	1	A			10
		2		A		10
		3			A	10
	A, B	4	A	B		10
		5	A		B	10
		6		A	B	10
		7	A, B			10
		8		A, B		10
		9			A, B	10
	A, B, C	10	A, B, C			10

**Table 4:** Experimental scheme for insufficient light conditions (14 lux, 2 lux).

Illuminance	Experimental Group Number	Forward Fall	Backward Fall	Sideways Fall	The Number of Experiments
14 lux	11	A	B		10
	12	A		B	10
	13		A	B	10
	14	A, B			10
	15		A, B		10
	16			A, B	10
2 lux	17	A	B		10
	18	A		B	10
	19		A	B	10
	20	A, B			10
	21		A, B		10
	22			A, B	10

**Table 5:** Indicators of complexity and size of reaction models.

Improvement Scheme	GPU Consumption	Parameter Quantity	GFLOPs	Training Duration
YOLOv7	2.51 G	37,218,132	105.2	17 h
YOLOv7 + CA	2.54 G	37,969,872	119.7	21 h
YOLOv7 + DO-DConv	2.52 G	36,672,340	104.7	17 h
YOLOv7 + Slim-Neck	2.48 G	34,246,516	38.5	14 h
YOLOv7 + DO-DConv + Slim-Neck	2.46 G	33,113,460	38.2	13 h

**Table 6:** Test results of each experimental scheme.

Fall Type	Precision	Recall	mAP@0.5	mAP@0.5:0.95	F1 Score	FPS
Forward Fall	86.00%	84.50%	85.00%	53.20%	85.20%	63
Backward Fall	85.30%	83.20%	84.40%	52.50%	84.20%	62
Sideways Fall	87.20%	85.80%	86.10%	54.80%	86.50%	65
Average	86.17%	84.50%	85.20%	53.50%	85.30%	63.3

**Table 7:** Test results under varying lighting conditions.

Lighting Condition	Fall Type	Precision	Recall	mAP@0.5	mAP@0.5:0.95	F1 Score	FPS
14 lux	Forward Fall	81.20%	79.60%	80.50%	50.30%	80.40%	58
	Backward Fall	80.40%	78.30%	79.00%	48.50%	79.40%	57
	Sideways Fall	82.10%	80.40%	81.20%	51.10%	81.20%	59
2 lux	Forward Fall	79.60%	77.10%	78.30%	47.10%	78.40%	56
	Backward Fall	78.20%	75.90%	77.00%	46.00%	76.90%	55
	Sideways Fall	80.50%	78.00%	79.30%	48.20%	79.30%	57

**Table 8:** The results of non-fall behavior recognition.

Action	Precision	Recall	F1 Score	False Alarm Rate
Walking	96.70%	95.30%	96.00%	3.00%
Running	95.20%	93.80%	94.50%	3.50%
Standing	97.30%	96.50%	96.90%	1.50%
Squatting	97.60%	96.70%	97.10%	1.80%

**Table 9:** Performance parameters of the model training.

Improvement Scheme	Precision	Recall Rate	mAP@0.5	mAP@0.5:0.95	FPS	F_1_ Score
YOLOv4-tiny	76.47%	83.11%	81.90%	51.06%	149.6	0.79
YOLOv5	82.63%	80.18%	84.90%	53.56%	130.2	0.81
YOLOv8n	87.93%	80.21%	89.75%	57.59%	113.7	0.84
YOLOv7	81.59%	83.81%	85.69%	54.31%	67	0.83
YOLOv7 + CA	71.58%	80.83%	78.76%	48.46%	57	0.76
YOLOv7 + DO-DConv	86.04%	82.84%	86.76%	55.63%	67	0.84
YOLOv7 + Slim-Neck	85.57%	85.57%	86.51%	54.25%	113	0.84
YOLOv7 + DO-DConv + Slim-Neck	88.03%	82.32%	88.10%	56.24%	114	0.86

**Table 10:** Confidence scores for different numbers of participants in a single experiment.

Experimental Group Number	First Second	Second Second	Third Second	Fourth Second
	Experimental Participant Numbers and Their Confidence Scores			
1	A: 0.71	A: 0.77	A: 0.84	A: 0.92
2	A: 0.52	A: 0.69	A: 0.76	A: 0.89
3	A: 0.69	A: 0.78	A: 0.82	A: 0.89
4	A: 0.74 B: 0.74	A: 0.63 B: 0.63	A: 0 B: 0	A: 0.88 B: 0.88
5	A: 0.81 B: 0.72	A: 0 B: 0.69	A: 0.69 B: 0.6	A: 0.91 B: 0.91
6	A: 0.77 B: 0.75	A: 0.6 B: 0.6	A: 0 B: 0.61	A: 0.84 B: 0.84
7	A: 0.78 B: 0.64	A: 0.71 B: 0.58	A: 0.64 B: 0.92	A: 0.91 B: 0.71
8	A: 0.86 B: 0.86	A: 0.78 B: 0.78	A: 0 B: 0.65	A: 0.81 B: 0.82
9	A: 0.7 B: 0.64	A: 0.54 B: 0	A: 0.61 B: 0.66	A: 0.71 B: 0.71
10	A: 0.78 B: 0.77 C: 0	A: 0.73 B: 0.63 C: 0	A: 0.47 B: 0.47 C: 0.27	A: 0.61 B: 0.64 C: 0.37

**Table 11:** Confidence levels at different brightness levels based on illumination intensity during the single experiment.

Experimental Group Number	First Second	Second Second	Third Second	Fourth Second
	Experimental Participant Numbers and Their Confidence Scores			
11	A: 0.62 B: 0	A: 0.63 B: 0.51	A: 0 B: 0	A: 0.79 B: 0.69
12	A: 0 B: 0.56	A: 0 B: 0	A: 0.5 B: 0.7	A: 0.75 B: 0.63
13	A: 0.75 B: 0.75	A: 0.81 B: 0.81	A: 0.72 B: 0.8	A: 0.71 B: 0.78
14	A: 0.65 B: 0.74	A: 0.66 B: 0.57	A: 0.86 B: 0.68	A: 0.76 B: 0.61
15	A: 0.69 B: 0.51	A: 0.56 B: 0.58	A: 0.7 B: 0.65	A: 0.81 B: 0.75
16	A: 0.71 B: 0.58	A: 0.73 B: 0.63	A: 0.82 B: 0.69	A: 0.87 B: 0.87
17	A: 0.56 B: 0.47	A: 063 B: 073	A: 0.77 B: 0.7	A: 0.82 B: 075
18	A: 0.54 B: 0.4	A: 0.73 B: 0.63	A: 0.3 B: 0	A: 0.75 B: 0.51
19	A: 0.61 B: 0.52	A: 0.28 B: 0.49	A: 0.52 B: 0.42	A: 0.71 B: 0.69
20	A: 0.47 B: 0.45	A: 0.4 B: 0.4	A: 0.6 B: 0.6	A: 0.74 B: 0.74
21	A: 0.62 B: 0.54	A: 0.71 B: 0.58	A: 0.44 B: 0.46	A: 0.7 B: 0.63
22	A: 0.51 B: 0	A: 0.6 B: 0.5	A: 0.56 B: 0.52	A: 0.7 B: 0.7

## Data Availability

Due to confidentiality agreements in our lab, we are unable to share the raw data at this time. However, we have provided comprehensive descriptions of the experimental design, analysis process, and results. We are happy to address any specific concerns.
